# Examining the effect of mitochondrial DNA variants on blood pressure in two Finnish cohorts

**DOI:** 10.1038/s41598-020-79931-6

**Published:** 2021-01-12

**Authors:** Jaakko Laaksonen, Pashupati P. Mishra, Ilkka Seppälä, Leo-Pekka Lyytikäinen, Emma Raitoharju, Nina Mononen, Maija Lepistö, Henrikki Almusa, Pekka Ellonen, Nina Hutri-Kähönen, Markus Juonala, Olli Raitakari, Mika Kähönen, Jukka T. Salonen, Terho Lehtimäki

**Affiliations:** 1 Department of Clinical Chemistry, Fimlab Laboratories and Finnish Cardiovascular Research Center Tampere, Faculty of Medicine and Health Technology, Tampere University, Arvo Ylpön katu 34, PO Box 100, 33014 Tampere, Finland; 2 Institute for Molecular Medicine (FIMM), University of Helsinki, Helsinki, Finland; 3 Department of Paediatrics, Tampere University Hospital and Faculty of Medicine and Health Technology, Tampere University, Tampere, Finland; 4 Department of Medicine, University of Turku, Turku, Finland; 5 Division of Medicine, Turku University Hospital, Turku, Finland; 6 Murdoch Children’s Research Institute, Parkville, VIC Australia; 7 Centre for Population Health Research, University of Turku and Turku University Hospital, Turku, Finland; 8 Research Centre for Applied and Preventive Cardiovascular Medicine, University of Turku, Turku, Finland; 9 Department of Clinical Physiology and Nuclear Medicine, University of Turku and Turku University Hospital, Turku, Finland; 10 Department of Clinical Physiology, Tampere University Hospital and Finnish Cardiovascular Research Center Tampere, Faculty of Medicine and Health Technology, Tampere University, Tampere, Finland; 11 Department of Public Health, Faculty of Medicine, University of Helsinki, Helsinki, Finland; 12 MAS-Metabolic Analytical Services Oy, Helsinki, Finland

**Keywords:** Genome-wide association studies, Hypertension, Mitochondrial genome

## Abstract

High blood pressure (BP) is a major risk factor for many noncommunicable diseases. The effect of mitochondrial DNA single-nucleotide polymorphisms (mtSNPs) on BP is less known than that of nuclear SNPs. We investigated the mitochondrial genetic determinants of systolic, diastolic, and mean arterial BP. MtSNPs were determined from peripheral blood by sequencing or with genome-wide association study SNP arrays in two independent Finnish cohorts, the Young Finns Study and the Finnish Cardiovascular Study, respectively. In total, over 4200 individuals were included. The effects of individual common mtSNPs, with an additional focus on sex-specificity, and aggregates of rare mtSNPs grouped by mitochondrial genes were evaluated by meta-analysis of linear regression and a sequence kernel association test, respectively. We accounted for the predicted pathogenicity of the rare variants within protein-encoding and the tRNA regions. In the meta-analysis of 87 common mtSNPs, we did not observe significant associations with any of the BP traits. Sex-specific and rare-variant analyses did not pinpoint any significant associations either. Our results are in agreement with several previous studies suggesting that mtDNA variation does not have a significant role in the regulation of BP. Future studies might need to reconsider the mechanisms thought to link mtDNA with hypertension.

## Introduction

High blood pressure (BP) is a major public health problem. It is an established risk factor for many noncommunicable diseases, such as cardiovascular disease, renal dysfunction, and dementia, and causes over 9 million premature deaths globally per year^1^. It is generally accepted that the regulation of BP is a multi-factorial trait, involving lifestyle, environmental, and genetic factors. To date, the majority of the genetic variants have been identified in studies of the nuclear genome^2–4^, and a limited number of studies have explicitly investigated the associations with variation in the mitochondrial genome. This gap in genetic knowledge is of a particular interest because, in addition to cellular energy production, mitochondria modulate, for example, the intracellular dynamics of nitric oxide, reactive oxygen species, and Ca^2+^, which, in turn, control endothelial function in blood vessels^5^.

Mitochondrial DNA (mtDNA) is a maternally inherited, double-stranded circular molecule containing 16,569 nucleotides that encode 22 transfer RNAs (tRNAs), two ribosomal RNAs (rRNAs), and 13 protein subunits of the four oxidative phosphorylation (OXPHOS) complexes^6^. The majority of the proteins functioning in the mitochondria are, however, encoded in nuclear DNA. There are multiple copies of mtDNA within each cell, and the mtDNA mutation rate is significantly higher than that of nuclear DNA^7^. Given the high mutation rate and the fact that both common and rare variants may influence the disease phenotypes^8^, the most appropriate method to study mtDNA is through sequencing. One possible mechanism through which mutations in the mtDNA contribute to BP variation is the oxidative stress due to an increased production of reactive oxygen species, which in turn causes cardiovascular and renal damage^9–11^. In addition to mutations in the mtDNA, the mitochondrial dysfunction may also be the consequence of mutations in the nuclear-encoded mitochondrial genes, or result from environmental and lifestyle factors.

Several studies with varying ethnic groups have demonstrated that a mutational hot spot for mitochondrial single-nucleotide polymorphisms (mtSNPs) associated with hypertension is in the tRNA-encoding genes^10,12,13^. However, many of these studies have been conducted on related individuals, and it is likely that most of these hypertension-associated variants are inherited and rare on a population level. While the role of naturally occurring mtDNA variation is still incompletely understood^14^, some evidence for the association of mtSNPs with BP exists from well-established cohorts. In the Framingham Heart Study, one mtSNP in OXPHOS complex IV correlated with systolic blood pressure (SBP)^15^. In a recent two-cohort sequencing study of older North American adults, both common and rare mtSNPs in the tRNA region were associated with variation in SBP in white participants^16^. Negative results have also been reported: 64 tagging mtSNPs that efficiently capture common mtDNA variation in the European population were not associated with BP in a study consisting of over 2000 individuals^17^, and a sequencing study conducted in South African population which utilized the MutPred pathogenicity prediction scores did not find a significant role for mtDNA variation in association with blood pressure levels^18^. A lexical tree analysis with phylogenetically related mtDNA variants in European population identified significant relationships with some common diseases, e. g. multiple sclerosis, but not with hypertension^19^.

The goal of the present study was to investigate the effect of mtDNA variants on SBP, diastolic blood pressure (DBP), and mean arterial pressure (MAP) among participants of two independent Finnish cohorts. We sought to both discover new and replicate the previously reported mitochondrial genetic determinants of BP. We studied both the effects of common single mtSNPs and the pooled effects of rare variants across seven mtDNA regions.

## Materials and methods

### Study participants

The Young Finns Study (YFS, http://youngfinnsstudy.utu.fi) is a Finnish longitudinal population study on the evolution of cardiovascular risk factors from childhood to adulthood^20^. We used the phenotype data from the follow-up in 2011 which included 2060 participants in total. The blood samples for mtDNA sequencing were obtained during the 2007 follow-up, when 2204 participants were examined. The study was approved by the ethical committee of the Hospital District of Southwest Finland (ETMK:68/1801/2017), and the study protocol of each study phase corresponded with the proposal by the World Health Organization.

The Finnish Cardiovascular Study (FINCAVAS) participant pool consists of patients recruited during 2001–2007 who underwent exercise stress tests at Tampere University Hospital^21^. A total of 4,068 participants completed a technically successful exercise test. The main indications for the exercise test were a suspicion of coronary heart disease (frequency 46%), the evaluation of work capacity (26%), testing for vulnerability to arrhythmia during exercise (25%), and assessing the adequacy of coronary heart disease treatment (13%); some patients had more than one indication. The study protocol was approved by the Ethical Committee of the Pirkanmaa Hospital District, Finland (R00153).

All participants in both study cohorts gave their written informed consent, and the studies were conducted in accordance with the Declaration of Helsinki.

### Blood pressure and other clinical measurements

In the YFS, BP was determined as the average of three measurements taken at two-minute intervals in a sitting position from the right arm brachial artery with a random zero sphygmomanometer. Korotkoff's first phase was used as the sign of SBP and the fifth phase as the sign of DBP. In the FINCAVAS, the patients lay in the supine position for 10 min, after which BP was measured once by an experienced nurse using a brachial cuff according to the Korotkoff’s method.

In both cohorts, the observed BPs were adjusted for antihypertensive medication usage. The medications were self-reported by the study participants, the duration of treatment was not known, and adherence was not assessed. Adjusted SBP was calculated by increasing the recorded measure by 8, 14, and 20 mmHg for 1, 2, and ≥ 3 medication classes taken, respectively. DBP measurements were adjusted similarly by increasing the recorded measure by 4, 10, and 16 mmHg for 1, 2, and ≥ 3 medication classes taken, respectively^22^. This adjustment method has proven to work with both simulated and real-life data^23^, and it maximizes the genetic and shared environmental variance components while minimizing individual-specific components^22^. FINCAVAS participants displayed clearly more antihypertensive medication usage (31%, 24%, and 16% on 1, 2, and ≥ 3 medication classes taken, respectively) than YFS participants (7%, 3%, and 0.3%, respectively). The mean adjustment was 10/6 and 13/9 mmHg for treated YFS and FINCAVAS participants, respectively. Adjusted MAP was calculated from the adjusted SBP and DBP values as MAP = DBP + 0.333 × (SBP—DBP).

Height and weight were measured, and body mass index (BMI) was calculated as weight in kilograms divided by height in meters squared.

### MtDNA sequencing and quality control in the YFS

Genomic DNA sample (n = 1807) concentrations were measured from whole-blood samples with the Qubit BR dsDNA kit (Life Technologies). MtDNA was amplified from the genomic DNA using the REPLI-g mtDNA kit (Qiagen) in a 50 μl reaction volume. The primer sites in the REPLI-g kit have been previously validated^24^. After the amplification mtDNA samples were processed into Illumina sequencing-compatible libraries with the Nextera DNA sample preparation kit (Illumina). The mtDNA concentrations were measured with Qubit dsDNA for Nextera tagmentation reaction. The reaction volume in the Nextera tagmentation and amplification steps was 20 μl, and after both steps the libraries were purified with an EdgeBio Performa V3 96-Well Short Plate (Edge BioSystems). After the amplification, the libraries were first incubated with 4 μl of EdgeBio SOPE resin and then purified with EdgeBio Performa plates. After purification, 48 samples with different index tags were pooled together (2 μl each) in each pool and concentrated with DNA Clean & Concentrator-5 (Zymo Research). The final volume of the concentrated pool was 15 μl. The sequencing-ready libraries were quantitated with an Agilent 2100 Bioanalyzer High Sensitivity kit (Agilent). The libraries were sequenced with the Illumina HiSeq system. All samples (n = 1658) that achieved any mean bait coverage were included in the further processing steps.

Paired-end FASTQ files were aligned with the revised Cambridge Reference Sequence (rCRS)^6^ using the sequence aligner BWA-MEM v. 0.7.17^25^. Reads mapped to the rCRS were sorted according to the start position and written into a BAM file using SAMtools v. 1.8^26^. Homo- and heteroplasmic variants were then detected with Mutserve v. 1.2.1, a stand-alone version of the web tool mtDNA-server^27^. We applied the default thresholds for mapping, base, and alignment quality scores in the Phred scale: 20, 20, and 30, respectively. Mutations in the mtDNA can affect either all or a varying proportion of the mtDNA molecules, and the terms used for these phenomena are homoplasmy and heteroplasmy, respectively^7^. The minimum heteroplasmy level was set to 0.05—we defined sites with a heteroplasmy level below this threshold as homoplasmic wild-type alleles and sites with a heteroplasmy level above 0.95 as homoplasmic variants. Mutserve applies a Bayesian model to the detection of homoplasmic variants, and in order to call a wild-type allele, we required a minimum sequencing coverage of five on both the forward and reverse strands. Mutserve identified mtSNPs in 1,365 different nucleotide positions from 1657 samples. Mean coverages per sample and per mtSNP were 497 and 525, respectively. With this coverage, the 0.05 heteroplasmy detection level is quite reliable^28^. Genotypes for heteroplasmic variants overlapping with any mtDNA-like sequence in the nucleus (NUMTs) were set to missing. The list of NUMTs insertions was based on the work by Dayama et al.^29^ We required each sequenced sample to have an overall mean coverage of ≥ 50, and 1,434 samples had a mean coverage above this threshold. Then, samples that did not have both phenotype and mtDNA data available were excluded, after which 1,150 samples remained for further analysis. After this, mean coverages per sample and per mtSNP were 563 and 568, respectively. Of the 1,365 identified common and rare mtSNPs, 249 had an allele frequency of ≥ 0.01. Heteroplasmy level between 0.05 and 0.95 in at least 1% of the samples was observed only for one mtSNP, m.16192C>T in the hypervariable region. This mtSNP was not genotyped in the FINCAVAS arrays and hence not included in the meta-analysis. In general, heteroplasmy rate was low in most sites, for 99% of the sites the number of heteroplasmic samples was three or less. This finding is in line with a previous study which found the incidence of heteroplasmy to be higher in tissues with high metabolic activity^30^.

### MtDNA genotyping and quality control in the FINCAVAS

Genomic DNA was extracted from peripheral blood leukocytes by using the QIAamp DNA Blood Minikit and automated biorobot M48 extraction (Qiagen). We applied the Illumina Cardio-MetaboChip and HumanCoreExome-12 v1.1 SNP arrays for genotyping mtSNPs. Genotyping was completed for 2,824 and 1,032 samples using the Cardio-MetaboChip and HumanCoreExome arrays, respectively. Genotypes were called using Illumina’s GenomeStudio GenCall algorithm. Samples with call rate of < 0.95, excess heterozygosity, cryptic relatedness (pi-hat > 0.2) and sex mismatch, as well as genetic outliers based on multi-dimensional scaling (MDS) plots, were removed. mtSNPs with a call rate of < 0.95 and a Hardy–Weinberg equilibrium *p*-value of ≤ 10^–6^ were also removed. Heterozygous genotypes possibly due to mitochondrial heteroplasmy or technical error were coded as missing. Homozygosity at a genotyped mtSNP indicates genotype frequency close to zero or one. After quality control, 53 mtSNPs from 2273 samples genotyped with the Cardio-MetaboChip array and 146 mtSNPs from 926 samples genotyped with the HumanCoreExome array were available. For four individuals that were genotyped with both arrays, the genotypes for the 34 overlapping mtSNPs were set as missing in the Cardio-MetaboChip array. After including only samples with both phenotype and mtDNA data available, 2193 and 923 samples from the Cardio-MetaboChip and HumanCoreExome arrays, respectively, were available for association analyses. The total number of individuals was 3112 since four samples were genotyped with both arrays. Of the variants genotyped with these arrays, 36 Cardio-MetaboChip mtSNPs and 67 HumanCoreExome mtSNPs had an allele frequency of ≥ 0.01.

### Statistical analyses

#### Association analyses of common variants

In order to investigate the associations of SBP, DBP, and MAP with mtDNA variants, BP levels were modeled as a linear function of the presence (coded as 1) or absence (coded as 0) of the variant allele using the lm function in R. Heteroplasmic genotypes were set to missing. In order to achieve normality of the BP distributions, we applied the rescaled inverse normal transformation, i.e. multiplied the inverse normal transformed BP values by the standard deviation of the original trait values in each cohort. This strategy makes the distributions normal and controls the type I error, and restores the original scale of measurement and thus enhances the power of meta-analysis^31^. Inverse normal transformation also effectively deals with phenotypic outliers^32^. Age, sex, and BMI were added as covariates to the linear regression models and the *p*-values were calculated using a standard *F* test with one degree of freedom. Association analyses were performed separately for the three data sets, and a random-effect meta-analysis was then performed using GWAMA software^33^. Variants with an allele frequency of ≥ 0.01 were included. The total number of mtSNPs included in the meta-analysis was 87, of which 22 were present in YFS and in both FINCAVAS data sets. The number of mtSNPs present in YFS and only in the FINCAVAS participants genotyped with the HumanCoreExome array was 52, and 13 mtSNPs were present in YFS and only in participants genotyped with the Cardio-MetaboChip array. Using Matrix Spectral Decomposition (matSpDlite)^34^ with the method of Li and Ji^35^, we determined that 45 of the 87 mtSNPs represented an estimate of the number of independent genetic effects for mtDNA. This resulted in a Bonferroni-corrected significance level of 0.0011 (i.e. 0.05/45). In the correction for multiple testing, we did not account for the testing of three BP traits, since they were correlated (Pearson's correlation coefficients between adjusted SBP, DBP, and MAP values 0.78–0.96 in the YFS and 0.67–0.92 in FINCAVAS).

#### Study power

With a sample size of ~ 2000, meaning that an mtSNP with an allele frequency of ≥ 0.01 was present in the YFS and the smaller FINCAVAS data set, our single-variant analysis had ~ 80% power to detect an mtSNP that explains 2.5% of the variance in BP. When an mtSNP with an allele frequency of ≥ 0.01 was present in the YFS and both FINCAVAS data sets, our analysis had ~ 95% and ~ 80% power to detect mtSNPs explaining 2.5% and 1% of the variance, respectively^36^.

#### Sex-specific analyses

The power of sex-combined analysis (i.e. males and females analyzed together) is reduced when heterogeneity is present in the allelic effects between the sexes. We examined the possible heterogeneity by applying the same linear model as described above to males and females separately, and by applying sex-differentiated meta-analysis in GWAMA^33,37^. In total, 66 mtSNPs with an allele frequency of ≥ 0.01 in both sexes were included in the meta-analysis. A sex-differentiated *p*-value below the individual *p*-values for males and females is indicative of a significant association with both sexes. We also tested for sex-specific heterogeneity, which is equivalent to a formal test of interaction with sex. A significant heterogeneity *p*-value would suggest that there is a difference in the effect sizes between the sexes. By using the same Matrix Spectral Decompositon as above, we now determined that 33 of the 66 mtSNPs represented an estimate of the number of independent genetic effects for mtDNA. To account for the two sexes tested, the significance level was now defined as 0.05/33/2 = 7.6 × 10^–4^.

#### Rare-variant analyses

Standard methods used to test for common variant associations are underpowered for detecting associations with rare variants^38^. The power of haplogroup-based analysis may also be insufficient if potential causative variants at a same mtDNA site are scattered across divergent haplogroups. The most common approaches for the analysis of rare-variant associations are the burden test and the sequence kernel association test (SKAT)^38,39^. The former collapses the rare variants within a specified region, assuming that all variants are either deleterious or protective. The latter aggregates and tests the collective effects of rare variants within a region without assuming similar directionality or effect size for each variant. Therefore, SKAT is superior to burden test for analyzing regions where both risk and protective variants as well as noncausal variants may be present^39,40^ and it has been successfully applied for both sequenced and genotyped mtDNA data^16,41,42^.

We used Mitomap^43^ to cluster the variants into seven regions, including each of the four OXPHOS complexes (I, III, IV, and V), all rRNAs combined, all tRNAs combined, and control region and non-coding regions combined. Homoplasmic alleles were coded as 0 or 2, corresponding to the reference and variant allele, respectively. Heteroplasmic genotypes were introduced in a dosage matrix, similarly to imputed genotypes. The dosage was calculated as twice the heteroplasmy rate. We employed a SKAT meta-analysis with minor allele frequencies (MAFs) of ≤ 0.01 (T1) and ≤ 0.05 (T5) for SBP, DBP, and MAP, using the seqMeta package in R, with the default beta weights, and with age, sex, and BMI as covariates. Bonferroni-corrected statistical significance was defined as 0.0071 (i.e. 0.05/7).

In order to assess the functional relevance of non-synonymous variants, we predicted the pathogenicity of the identified variants with MutPred^44,45^ and MitoTIP^46^ pathogenicity scores. The MutPred algorithm assigns each variant in the protein-encoding mtDNA regions a pathogenicity score between 0 and 1. Variants with a MutPred score > 0.5 are potentially “harmful”, and variants with a score > 0.75 should be considered a high confidence “harmful”. MitoTIP predicts the pathogenicity of the variants in the tRNA regions, and the prediction scores have been interpreted within quartiles as “likely benign”, “possibly benign”, “possibly pathogenic” or “likely pathogenic”. We leveraged SKAT meta-analysis for the BP levels, similarly as described above, but including only variants with a MutPred score > 0.5 or a MitoTIP classification “possibly” or “likely pathogenic”.

#### Control for population stratification

The use of mtDNA principal components (PCs) as covariates has been demonstrated to be a robust method to adjust for population stratification in genetic association studies. In addition, the use of mitochondrial PCs effectively removes false-positive associations but does not cause a loss of power in detecting true associations^47,48^. Logistic PC analysis was performed on all homoplasmic genotypes passing quality control and with a MAF of ≥ 0.01 using the logisticPCA package^49^. For each data set, we selected the number of mitochondrial PCs for the single variant analyses so that the median chi-squared-based genomic inflation factor (λ_GC_) was as close to one as possible. For SKAT, we selected the same number of PCs as were used in the sex-combined common variant analyses. Values of λ_GC_ < 1.05 are generally considered benign^50^. For any result data sets with λ_GC_ > 1.05, genomic control was applied by multiplying the standard errors of regression coefficients by the square root of the inflation factor of the respective study.

## Results

On average, participants in the FINCAVAS were older and had higher BMI and BP levels than participants in the YFS (Table 1). We evaluated the associations of 87 common mtDNA variants with SBP, DBP, and MAP using random-effect meta-analysis in these two Finnish cohorts, with sample sizes of up to 4,262. When both sexes were analyzed together, we did not observe any statistically significant associations. The top three associations are shown in Table 2. All meta-analysis results and the quantile–quantile plots from the cohort-level analyses are provided in the Supplemental material, in Table S1 and Fig. S1.

**Table 1:** Baseline characteristics of the YFS and FINCAVAS cohorts. Values are means (SD).

	YFS n = 1150	FINCAVAS n = 3112
Age, years	42.2 (5.0)	56.3 (13.0)
Women, n (%)	661 (57.5)	1,214 (39.0)
Body mass index, kg/m^2^	26.5 (5.00)	27.5 (4.5)
Unadjusted SBP, mmHg	118.7 (13.9)	136.7 (18.9)
Unadjusted DBP, mmHg	74.8 (10.4)	80.0 (9.6)
Unadjusted MAP, mmHg	89.5 (10.8)	98.9 (11.3)
Medication-adjusted SBP, mmHg	119.7 (14.7)	145.6 (21.1)
Medication-adjusted DBP, mmHg	75.4 (10.8)	86.2 (11.2)
Medication-adjusted MAP, mmHg	90.2 (11.4)	106.0 (13.3)

*YFS* Young Finns Study, *FINCAVAS* Finnish Cardiovascular Study, *SBP* systolic blood pressure, *DBP* diastolic blood pressure, *MAP* mean arterial pressure.

**Table 2:** Three most significant associations in the sex-combined meta-analysis.

mtSNP	Trait	Beta (SE)	*p*	MAF	n
m.1243T>C	SBP	− 4.22 (1.79)	0.019	0.024	4,219
m.15257G>A	MAP	4.91 (2.20)	0.025	0.013	2,071
m.11674C>T	SBP	− 4.26 (1.91)	0.026	0.029	3,342

*mtSNP* mitochondrial single-nucleotide polymorphism, *Trait* the trait used for a specific test, *Beta (SE)* beta coefficient and the corresponding standard error, *p* unadjusted *p*-value from meta-analysis, *MAF* minor allele frequency, *n* sample size contributing in a particular mtSNP meta-analysis.

There was no evidence for differences between the sexes in the mitochondrial genetic control of BP levels. All results from the sex-differentiated and heterogeneity meta-analysis as well as the quantile–quantile plots from the cohort-level analyses are provided in the Supplemental material, in Table S2 and Figs. S2–S3.

Finally, we conducted SKAT meta-analyses on all rare (T1 test) and low-frequency (T5 test) variants. We also employed SKAT taking into account the predicted pathogenicity of the variants. None of the analyses yielded significant associations with the BP traits over of the tested mtDNA regions. All results from SKAT meta-analysis are provided in the Supplemental material, Tables S3 and S4.

## Discussion

We conducted one the largest studies to date investigating the possible mitochondrial genetic determinants of BP. We did not find any associations that survived after correction for multiple testing.

We could not replicate the results where the common mtSNPs m.3197T>C and m.15924A>G were associated with higher SBP and MAP, respectively, in white North American individuals^16^. In the current study, the former mtSNP was sequenced or genotyped in over 2000 and the latter in over 4200 individuals, which casts doubt over the role of these two mtSNPs in the variation of BP. Possible confounding effects due to different genetic ancestry should not have had an effect on the associations, since both the previous and the current study included mitochondrial PCs as regression covariates^47,48^. However, it should be noted that, since the North American individuals were significantly older than the Finns in our cohorts, it is possible that the regulative role of these two mtSNPs is activated only in later life.

The aforementioned North American study^16^ identified significant pooled effects on SBP across variants in the tRNA regions in white participants. Our analysis of rare and low-frequency variants did not yield any significant associations, not even when we accounted for the predicted pathogenicity of the variants within protein-encoding and the tRNA regions. We did not account for the functional relevance of the variants in the rRNA regions because to the best of our knowledge, no tools exist for annotating pathogenicity for variants in those regions.

Another mtSNP previously found to be associated with SBP is m.5913G>A in the Framingham Heart Study^15^. In the current study, this mtSNP was only sequenced in the YFS population with a MAF of < 0.01, while it was not genotyped in FINCAVAS, which left us unable to study the effect of this mtSNP in single-variant analysis. While the sample size of the current study is, to the authors' knowledge, one of largest used in investigating mtDNA associations with BP, our inability to replicate earlier findings from well-characterized cohorts underlines the need for mtDNA association studies with significantly larger sample sizes from multiple cohorts. For example, a recent study including ~ 170,000 individuals from 45 cohorts reported associations with seven metabolic outcomes, but these did not include BP^42^. These large consortium studies can however be extremely costly, and another basis for future research could be achieved by introducing more homogeneous study groups with less confounding effects. We should also be ready to accept to the null hypothesis that mtDNA variants do not contribute to BP variation on a significant level as it is implied by several previous studies^17–19^. Another hypothesis to be tested is that instead of being causal of BP variation, the mtDNA variants would impact the hypertension complications or alter the course of the disease^51^.

The strength of the current study was that the mtSNPs in the YFS were obtained through sequencing, which allowed us to study the effects of both common and rare variants. Another strength was the large range of blood pressure variation in our two independent cohorts, and the method we used to adjust for antihypertensive treatment effects has proven to work across a wire variety of clinical scenarios^22,23^. Some limitations should also be acknowledged—the mtSNPs in FINCAVAS were genotyped, resulting in a relatively small number of mtSNPs included in the meta-analyses. The majority of the rare mtSNPs were identified only in the YFS population which decreased the power of the SKAT meta-analysis. We also applied a stringent threshold to define homoplasmic variants and wild-type alleles, but relaxing the threshold to e. g. 10% and 90% would have increased the number of homoplasmic alleles only marginally. Another limitation is that we only investigated substitutions, whereas mtDNA deletions and insertions were not identified. In addition, BP measurements were performed only once for the FINCAVAS participants. Ambulatory BP monitoring could greatly increase the robustness of the future studies.

In conclusion, we found no support for the hypothesis that common or rare mtSNPs play a significant role in the regulation of BP. While studies with larger sample sizes might show different results, we should also be open to the idea that the non-significant outcomes reported by the current and previous studies are in fact correct, and that future studies concerning this topic need to reconsider the mechanisms thought to link mtDNA with hypertension.

## Supplementary Information

Supplementary Information.

## Data Availability

The data sets generated and analyzed during the current study comprise health-related participant data, and their use is therefore restricted under the regulations concerning professional secrecy (Act on the Openness of Government Activities, 612/1999) and sensitive personal data (Personal Data Act, 523/1999, implementing the EU data protection directive 95/46/EC). Due to these legal constraints, the Ethics Committee of the Hospital District of Southwest Finland has stated in 2016 that individual-level data cannot be stored in public repositories or otherwise made publicly available. Data are, however, available from the authors upon a reasonable request.
