# Supplementary material for: Examining the effect of mitochondrial DNA variants on blood pressure in two Finnish cohorts
Source: Sci Rep. 2021 Jan 12;11:611. doi: 10.1038/s41598-020-79931-6 (PMC7804469; doi:10.1038/s41598-020-79931-6)
Supplement: Supplementary file 1 — Supplementary Information. [file 41598_2020_79931_MOESM1_ESM.pdf]

Examining the effect of mitochondrial DNA variants on blood pressure in two Finnish cohorts

Jaakko Laaksonen[1*], Pashupati P. Mishra[1], Ilkka Seppälä[1], Leo-Pekka Lyytikäinen[1], Emma Raitoharju[1], Nina Mononen[1], Maija Lepistö[2], Henrikki Almusa[2], Pekka Ellonen[2], Nina Hutri-Kähönen[3], Markus Juonala[4,5,6], Olli Raitakari[7,8,9], Mika Kähönen[10], Jukka T. Salonen[11,12], Terho Lehtimäki[1]

[1]Department of Clinical Chemistry, Fimlab Laboratories and Finnish Cardiovascular Research Center Tampere, Faculty of Medicine and Health Technology, Tampere University, Tampere, Finland

[2]Institute for Molecular Medicine (FIMM), University of Helsinki, Helsinki, Finland

[3]Department of Paediatrics, Tampere University Hospital and Faculty of Medicine and Health Technology, Tampere University, Tampere, Finland

[4]Department of Medicine, University of Turku, Turku, Finland

[5]Division of Medicine, Turku University Hospital, Turku, Finland

[6]Murdoch Children's Research Institute, Parkville, Victoria, Australia

[7]Centre for Population Health Research, University of Turku and Turku University Hospital, Turku, Finland

[8]Research Centre for Applied and Preventive Cardiovascular Medicine, University of Turku, Turku, Finland

[9]Department of Clinical Physiology and Nuclear Medicine, University of Turku and Turku University Hospital, Turku, Finland

[10]Department of Clinical Physiology, Tampere University Hospital and Finnish Cardiovascular Research Center Tampere, Faculty of Medicine and Health Technology, Tampere University, Tampere, Finland

[11]Department of Public Health, Faculty of Medicine, University of Helsinki, Helsinki, Finland

[12]MAS-Metabolic Analytical Services Oy, Helsinki, Finland

*Correspondence: jaakko.h.laaksonen@tuni.fi

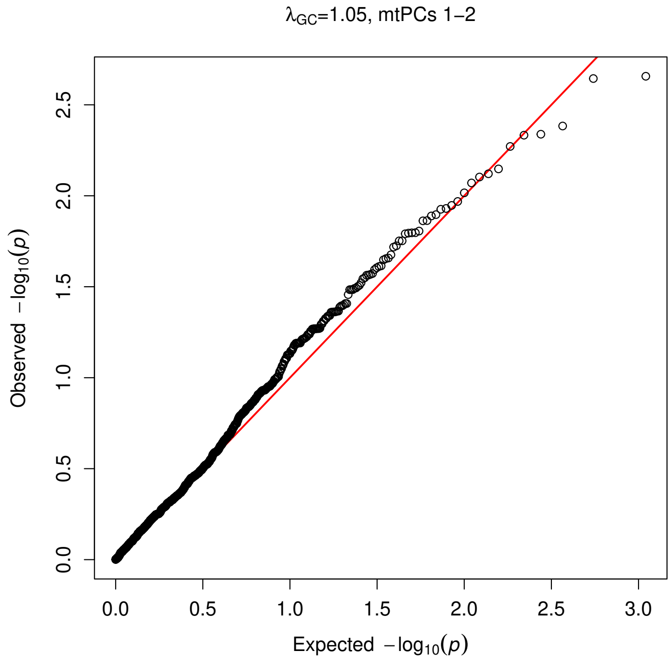

(a) YFS

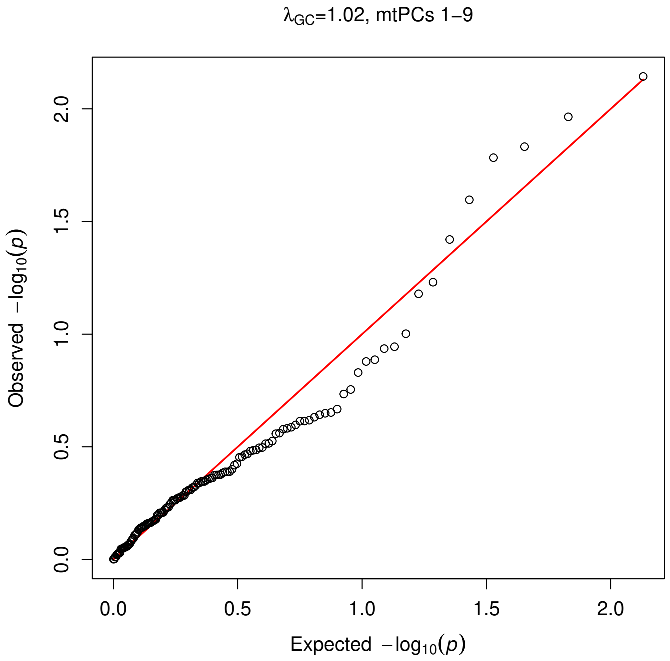

(b) FINCAVAS (MetaboChip array)

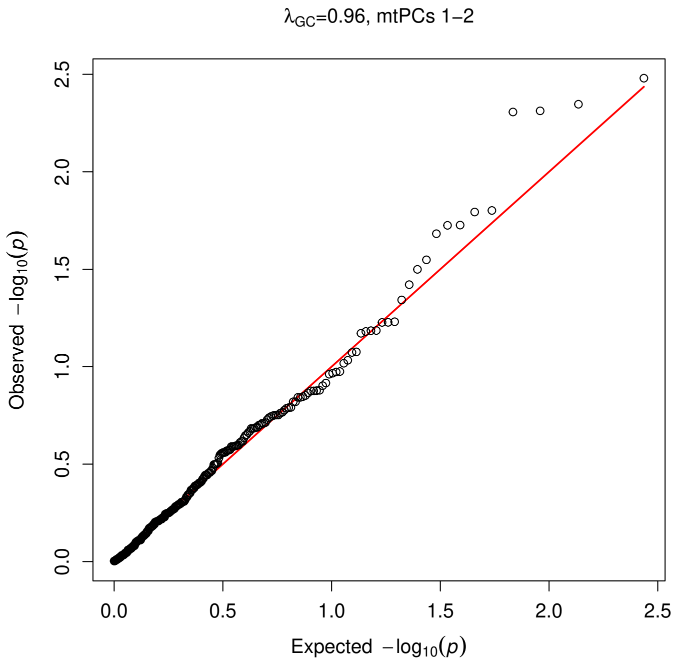

(c) FINCAVAS (CoreExome array)

Figure S1: QQ-plots of p-values from the sex-combined analysis in the individual data sets. Each header indicates the genomic inflation factor (λ_{GC}), and the number of principal components (mtPCs) used to correct for population stratification.

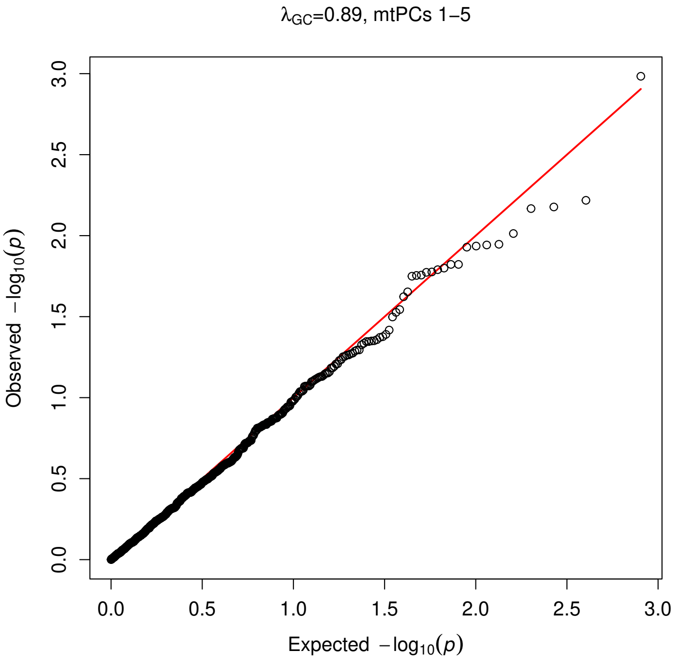

(a) YFS

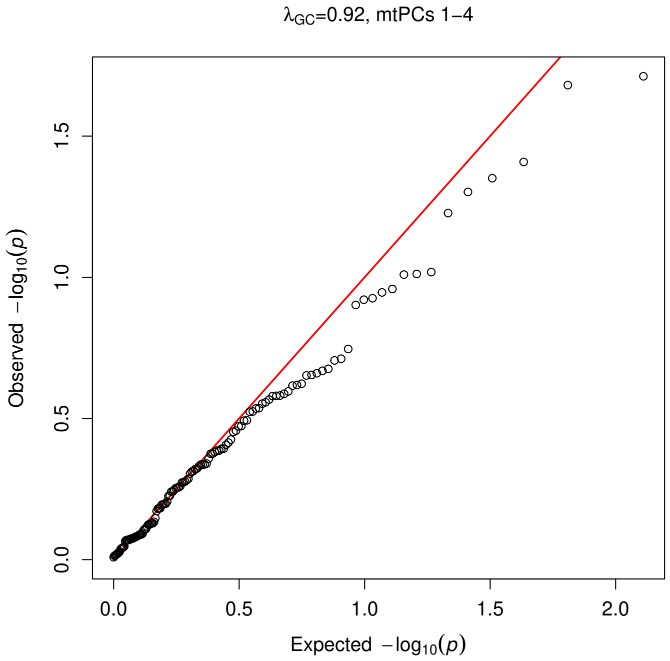

(b) FINCAVAS (MetaboChip array)

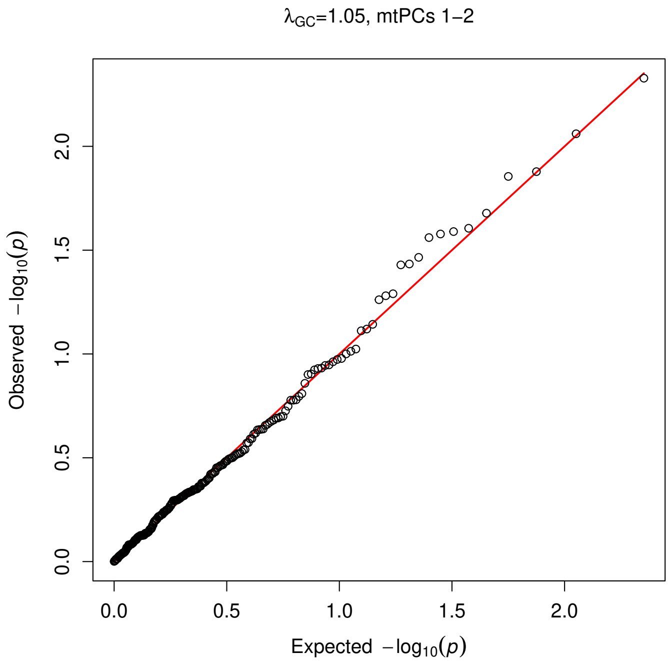

(c) FINCAVAS (CoreExome array)

Figure S2: QQ-plots of p-values from the male-specific analysis in the individual data sets. Each header indicates the genomic inflation factor (λ_{GC}), and the number of principal components (mtPCs) used to correct for population stratification.

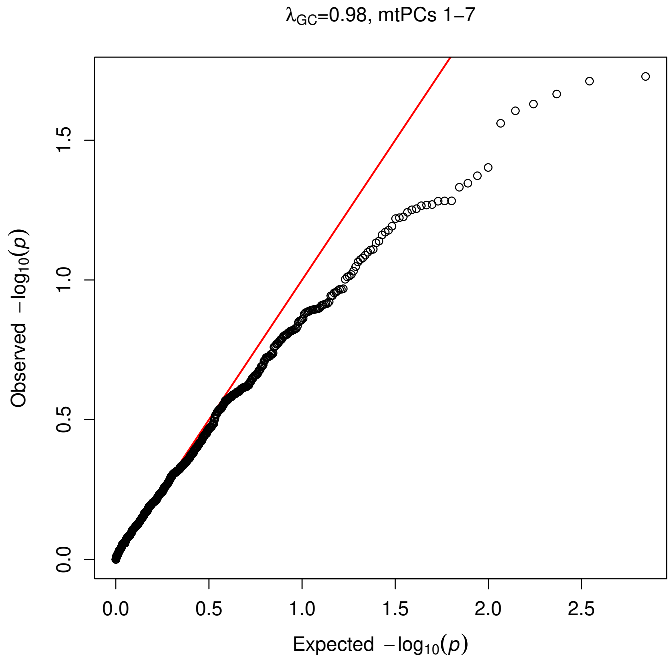

(a) YFS

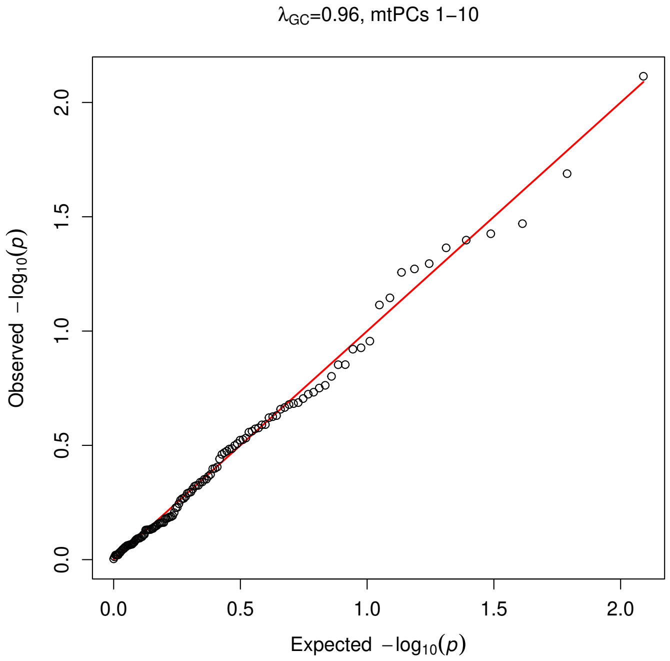

(b) FINCAVAS (MetaboChip array)

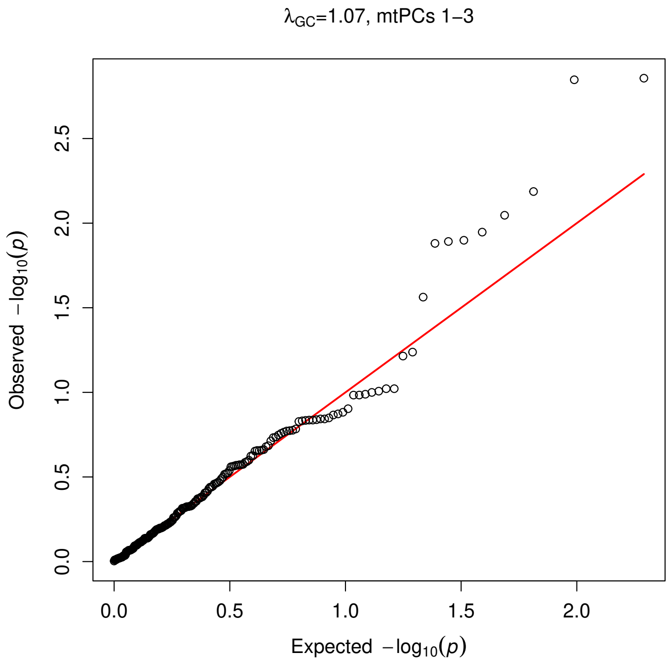

(c) FINCAVAS (CoreExome array)

Figure S3: QQ-plots of p-values from the female-specific analysis in the individual data sets. Each header indicates the genomic inflation factor (λ_{GC}), and the number of principal components (mtPCs) used to correct for population stratification.

Table S1. Meta-analysis results of the sex-combined analysis.

Trait	mtSNP position	Variant allele	Reference allele	VAF	Beta	SE	P	N
SBP	93	G	A	0.022	2.142	2.213	0.333	2041
MAP	93	G	A	0.022	-0.232	1.651	0.888	2041
DBP	93	G	A	0.022	-1.26	1.528	0.41	2041
MAP	199	C	T	0.034	2.596	1.4	0.064	2023
DBP	199	C	T	0.034	1.789	1.572	0.255	2023
SBP	199	C	T	0.034	3.418	1.92	0.075	2023
SBP	217	C	T	0.061	-0.633	1.545	0.682	2016
MAP	217	C	T	0.061	-0.948	1.14	0.406	2016
DBP	217	C	T	0.061	-0.957	1.052	0.363	2016
SBP	228	A	G	0.038	-1.882	1.884	0.318	2016
DBP	228	A	G	0.038	-2.082	1.292	0.107	2016
MAP	228	A	G	0.038	-2.262	1.398	0.106	2016
DBP	295	T	C	0.069	-1.16	1.078	0.282	1987
SBP	295	T	C	0.069	-0.138	1.545	0.929	1987
MAP	295	T	C	0.069	-0.853	1.163	0.463	1987
MAP	456	T	C	0.025	0.244	1.58	0.877	2008
DBP	456	T	C	0.025	-0.426	1.448	0.768	2008
SBP	456	T	C	0.025	1.637	2.169	0.45	2008
SBP	477	C	T	0.018	1.037	2.531	0.682	2014
MAP	477	C	T	0.018	0.38	1.865	0.839	2014
DBP	477	C	T	0.018	-0.247	1.714	0.886	2014
SBP	499	A	G	0.019	-6.897	8.714	0.429	2005
DBP	499	A	G	0.019	-0.644	2.218	0.772	2005
MAP	499	A	G	0.019	-2.587	4.503	0.566	2005
DBP	709	A	G	0.107	0.056	1.708	0.974	2039
SBP	709	A	G	0.107	-2.297	1.254	0.067	2039
MAP	709	A	G	0.107	-0.695	1.588	0.662	2039
MAP	951	A	G	0.031	-0.136	1.397	0.923	2032
DBP	951	A	G	0.031	-0.575	1.294	0.657	2032
SBP	951	A	G	0.031	1.011	1.87	0.589	2032
SBP	1243	C	T	0.024	-4.224	1.794	0.019	4219
DBP	1243	C	T	0.024	-0.118	1.815	0.948	4219
MAP	1243	C	T	0.024	-1.273	1.486	0.392	4219
MAP	1719	A	G	0.048	0.863	1.071	0.42	4211
SBP	1719	A	G	0.048	1.657	1.789	0.354	4211
DBP	1719	A	G	0.048	0.509	0.97	0.6	4211
MAP	1721	T	C	0.012	1.808	2.294	0.431	2042
SBP	1721	T	C	0.012	0.324	3.185	0.919	2042
DBP	1721	T	C	0.012	1.852	2.092	0.376	2042
MAP	1811	G	A	0.084	0.856	0.802	0.286	4172
DBP	1811	G	A	0.084	1.067	0.729	0.143	4172
SBP	1811	G	A	0.084	-0.005	1.118	0.996	4172
DBP	1888	A	G	0.051	0.224	1.251	0.858	4202
MAP	1888	A	G	0.051	0.636	1.353	0.638	4202
SBP	1888	A	G	0.051	2.064	1.822	0.257	4202
SBP	2259	T	C	0.017	-1.388	2.362	0.557	2042
MAP	2259	T	C	0.017	-1.306	1.815	0.472	2042
DBP	2259	T	C	0.017	-0.705	1.709	0.68	2042
SBP	2706	G	A	0.559	0.331	1.357	0.807	4223
DBP	2706	G	A	0.559	0.608	0.909	0.503	4223
MAP	2706	G	A	0.559	0.433	0.992	0.663	4223
MAP	3010	A	G	0.275	-0.441	0.618	0.476	2044
DBP	3010	A	G	0.275	-0.422	0.569	0.458	2044
SBP	3010	A	G	0.275	-0.363	0.837	0.665	2044

MAP	3116	T	C	0.012	0.118	2.224	0.958	2047
SBP	3116	T	C	0.012	-0.71	3.04	0.815	2047
DBP	3116	T	C	0.012	0.128	2.045	0.95	2047
SBP	3197	C	T	0.202	-0.348	2.186	0.873	2043
MAP	3197	C	T	0.202	-0.758	1.58	0.632	2043
DBP	3197	C	T	0.202	-0.762	1.443	0.597	2043
MAP	3434	G	A	0.017	-0.7	1.92	0.715	2044
SBP	3434	G	A	0.017	0.857	2.556	0.737	2044
DBP	3434	G	A	0.017	-1.153	1.784	0.518	2044
DBP	3480	G	A	0.048	2.386	1.48	0.107	3308
MAP	3480	G	A	0.048	1.949	1.564	0.213	3308
SBP	3480	G	A	0.048	0.999	2.001	0.618	3308
DBP	3720	G	A	0.015	1.571	1.847	0.395	2049
MAP	3720	G	A	0.015	1.748	2.649	0.509	2049
SBP	3720	G	A	0.015	1.403	5.091	0.783	2049
DBP	3915	A	G	0.012	-1.645	1.498	0.272	4232
MAP	3915	A	G	0.012	-1.692	1.696	0.318	4232
SBP	3915	A	G	0.012	-2.779	2.519	0.27	4232
SBP	4024	G	A	0.015	5.151	6.136	0.401	2040
DBP	4024	G	A	0.015	4.246	1.994	0.033	2040
MAP	4024	G	A	0.015	4.929	3.602	0.171	2040
MAP	4216	C	T	0.115	-0.474	1.24	0.702	2032
SBP	4216	C	T	0.115	1.603	1.614	0.321	2032
DBP	4216	C	T	0.115	-1.207	1.164	0.299	2032
MAP	4336	C	T	0.011	-4.095	1.963	0.037	3310
DBP	4336	C	T	0.011	-3.544	1.754	0.043	3310
SBP	4336	C	T	0.011	-5.025	2.809	0.074	3310
MAP	4769	G	A	0.958	-1.178	1.212	0.331	2058
SBP	4769	G	A	0.958	-2.848	1.636	0.082	2058
DBP	4769	G	A	0.958	-0.482	1.121	0.667	2058
DBP	4917	G	A	0.048	0.278	1.269	0.826	2032
MAP	4917	G	A	0.048	0.901	1.371	0.511	2032
SBP	4917	G	A	0.048	3.073	1.841	0.095	2032
SBP	5004	C	T	0.015	5.143	6.167	0.404	2035
MAP	5004	C	T	0.015	4.906	3.637	0.177	2035
DBP	5004	C	T	0.015	4.223	2.021	0.037	2035
DBP	5263	T	C	0.02	0.835	1.599	0.602	2034
SBP	5263	T	C	0.02	-0.968	2.334	0.678	2034
MAP	5263	T	C	0.02	0.116	1.732	0.947	2034
DBP	5390	G	A	0.017	0.659	2.058	0.749	2041
MAP	5390	G	A	0.017	0.86	2.545	0.736	2041
SBP	5390	G	A	0.017	0.392	3.963	0.921	2041
MAP	5460	A	G	0.038	-0.254	2.039	0.901	2033
DBP	5460	A	G	0.038	1.012	1.966	0.607	2033
SBP	5460	A	G	0.038	-3.485	2.385	0.144	2033
DBP	6734	A	G	0.011	1.892	2.146	0.378	2045
SBP	6734	A	G	0.011	0.326	3.058	0.915	2045
MAP	6734	A	G	0.011	1.374	2.306	0.551	2045
DBP	6776	C	T	0.019	-0.692	1.638	0.673	2024
SBP	6776	C	T	0.019	-2.487	2.243	0.267	2024
MAP	6776	C	T	0.019	-1.492	1.737	0.39	2024
SBP	7476	T	C	0.012	3.615	3.225	0.262	2036
DBP	7476	T	C	0.012	2.046	2.125	0.335	2036
MAP	7476	T	C	0.012	2.966	2.328	0.203	2036
DBP	7768	G	A	0.123	-0.703	1.75	0.688	2034
MAP	7768	G	A	0.123	-0.504	1.427	0.724	2034

SBP	7768	G	A	0.123	0.277	1.702	0.871	2034
DBP	8616	T	G	0.011	3.516	2.189	0.108	2059
MAP	8616	T	G	0.011	2.877	2.358	0.222	2059
SBP	8616	T	G	0.011	1.315	3.133	0.675	2059
SBP	8869	G	A	0.023	-0.103	2.17	0.962	2069
MAP	8869	G	A	0.023	0.237	1.606	0.882	2069
DBP	8869	G	A	0.023	0.687	1.482	0.643	2069
SBP	9055	A	G	0.054	1.228	1.764	0.486	3334
DBP	9055	A	G	0.054	2.569	1.263	0.042	3334
MAP	9055	A	G	0.054	2.097	1.351	0.121	3334
MAP	9477	A	G	0.189	-0.592	1.569	0.706	2062
DBP	9477	A	G	0.189	-0.491	1.432	0.732	2062
SBP	9477	A	G	0.189	-0.118	2.174	0.957	2062
SBP	9667	G	A	0.021	-0.617	2.674	0.818	2069
DBP	9667	G	A	0.021	-1.469	1.601	0.359	2069
MAP	9667	G	A	0.021	-1.356	1.752	0.439	2069
SBP	9698	C	T	0.059	0.055	1.442	0.97	4259
DBP	9698	C	T	0.059	0.635	1.194	0.595	4259
MAP	9698	C	T	0.059	0.685	1.034	0.508	4259
DBP	9899	C	T	0.01	0.819	2.123	0.7	4260
SBP	9899	C	T	0.01	2.452	2.741	0.371	4260
MAP	9899	C	T	0.01	1.136	1.906	0.551	4260
SBP	9903	C	T	0.022	-3.554	2.249	0.114	2071
DBP	9903	C	T	0.022	0.333	1.522	0.827	2071
MAP	9903	C	T	0.022	-1.101	1.654	0.506	2071
SBP	10034	C	T	0.025	3.276	2.638	0.214	4252
MAP	10034	C	T	0.025	2.597	1.637	0.113	4252
DBP	10034	C	T	0.025	2.061	1.493	0.167	4252
SBP	10238	C	T	0.028	3	2.09	0.151	4258
DBP	10238	C	T	0.028	2.058	1.381	0.136	4258
MAP	10238	C	T	0.028	2.465	1.513	0.103	4258
SBP	10463	C	T	0.053	1.904	1.717	0.267	4262
DBP	10463	C	T	0.053	0.655	1.177	0.578	4262
MAP	10463	C	T	0.053	0.81	1.275	0.525	4262
DBP	10550	G	A	0.049	1.168	1.22	0.339	4262
SBP	10550	G	A	0.049	0.66	1.637	0.687	4262
MAP	10550	G	A	0.049	1.078	1.182	0.362	4262
SBP	10754	G	A	0.012	1.842	3.066	0.548	2062
MAP	10754	G	A	0.012	-0.536	2.275	0.814	2062
DBP	10754	G	A	0.012	-2.213	3.344	0.508	2062
DBP	11299	C	T	0.048	2.462	1.452	0.09	3339
SBP	11299	C	T	0.048	3.252	4.657	0.485	3339
MAP	11299	C	T	0.048	2.138	1.534	0.163	3339
DBP	11467	G	A	0.283	1.808	2.711	0.505	2066
SBP	11467	G	A	0.283	0.791	1.897	0.677	2066
MAP	11467	G	A	0.283	1.65	2.339	0.481	2066
SBP	11674	T	C	0.029	-4.261	1.913	0.026	3342
MAP	11674	T	C	0.029	-2.203	1.483	0.137	3342
DBP	11674	T	C	0.029	-2.858	2.785	0.305	3342
SBP	11812	G	A	0.04	0.84	2.032	0.679	3341
DBP	11812	G	A	0.04	-1.274	1.417	0.368	3341
MAP	11812	G	A	0.04	-0.628	1.53	0.681	3341
SBP	11899	C	T	0.012	5.145	3.483	0.14	4249
MAP	11899	C	T	0.012	2.797	2.872	0.33	4249
DBP	11899	C	T	0.012	1.777	2.51	0.479	4249
SBP	11914	A	G	0.043	0.987	1.297	0.447	4248

DBP	11914	A	G	0.043	0.687	0.87	0.43	4248
MAP	11914	A	G	0.043	0.822	0.99	0.406	4248
DBP	12308	G	A	0.299	1.766	1.96	0.368	4246
SBP	12308	G	A	0.299	0.333	1.892	0.86	4246
MAP	12308	G	A	0.299	1.436	1.882	0.445	4246
SBP	12372	A	G	0.304	1.036	2.138	0.628	3328
DBP	12372	A	G	0.304	2.633	3.154	0.404	3328
MAP	12372	A	G	0.304	1.582	3.921	0.687	3328
DBP	12612	G	A	0.065	-0.456	1.267	0.719	3342
SBP	12612	G	A	0.065	0.783	1.692	0.644	3342
MAP	12612	G	A	0.065	-0.095	1.333	0.943	3342
DBP	12630	A	G	0.011	1.185	2.163	0.584	2073
SBP	12630	A	G	0.011	-2.014	3.05	0.509	2073
MAP	12630	A	G	0.011	-0.092	2.317	0.968	2073
MAP	12669	T	C	0.011	-0.603	2.346	0.797	2072
SBP	12669	T	C	0.011	-5.708	3.139	0.069	2072
DBP	12669	T	C	0.011	1.115	2.173	0.608	2072
SBP	13020	C	T	0.014	0.409	2.502	0.87	3329
MAP	13020	C	T	0.014	0.767	1.774	0.666	3329
DBP	13020	C	T	0.014	0.424	1.597	0.791	3329
SBP	13708	A	G	0.071	-0.193	1.46	0.895	4181
DBP	13708	A	G	0.071	-0.883	1.008	0.381	4181
MAP	13708	A	G	0.071	-0.682	1.089	0.531	4181
SBP	13759	A	G	0.026	2.789	3.969	0.482	2057
DBP	13759	A	G	0.026	0.295	2.226	0.894	2057
MAP	13759	A	G	0.026	1.017	2.746	0.711	2057
DBP	13928	C	G	0.026	1.068	1.195	0.372	3319
SBP	13928	C	G	0.026	0.424	1.836	0.817	3319
MAP	13928	C	G	0.026	0.95	1.32	0.472	3319
SBP	13966	G	A	0.017	0.909	3.186	0.775	4253
MAP	13966	G	A	0.017	0.033	2.363	0.989	4253
DBP	13966	G	A	0.017	-0.53	1.97	0.788	4253
SBP	14182	C	T	0.134	1.215	2.319	0.6	2052
MAP	14182	C	T	0.134	0.41	2.461	0.868	2052
DBP	14182	C	T	0.134	-0.011	2.453	0.996	2052
DBP	14233	G	A	0.042	0.388	1.717	0.821	2071
SBP	14233	G	A	0.042	2.065	1.902	0.277	2071
MAP	14233	G	A	0.042	0.805	1.657	0.627	2071
SBP	14766	T	C	0.507	10.69	14.045	0.447	3328
DBP	14766	T	C	0.507	1.233	2.093	0.556	3328
MAP	14766	T	C	0.507	5.233	6.336	0.409	3328
SBP	14905	A	G	0.054	8.367	10.575	0.429	3272
DBP	14905	A	G	0.054	7.618	10.678	0.476	3272
MAP	14905	A	G	0.054	8.246	10.878	0.448	3272
MAP	15043	A	G	0.039	1.428	1.283	0.265	4240
DBP	15043	A	G	0.039	1.203	1.081	0.266	4240
SBP	15043	A	G	0.039	1.972	2.234	0.377	4240
MAP	15218	G	A	0.026	-1.256	1.589	0.429	2062
DBP	15218	G	A	0.026	-0.483	1.46	0.741	2062
SBP	15218	G	A	0.026	-1.986	2.17	0.36	2062
SBP	15257	A	G	0.014	5.778	3.054	0.059	2071
MAP	15257	A	G	0.014	4.916	2.2	0.025	2071
DBP	15257	A	G	0.014	3.904	2.007	0.052	2071
DBP	15326	G	A	0.993	-2.35	2.713	0.386	2072
SBP	15326	G	A	0.993	-8.161	3.976	0.04	2072
MAP	15326	G	A	0.993	-4.244	2.941	0.149	2072

MAP	15452	A	C	0.116	-0.192	1.247	0.878	4254
SBP	15452	A	C	0.116	1.98	1.628	0.224	4254
DBP	15452	A	C	0.116	-0.989	1.168	0.397	4254
MAP	15607	G	A	0.053	-0.383	1.635	0.815	3338
DBP	15607	G	A	0.053	-0.342	3.219	0.915	3338
SBP	15607	G	A	0.053	1.862	2.075	0.37	3338
SBP	15693	C	T	0.018	-6.848	8.861	0.44	2070
DBP	15693	C	T	0.018	-0.589	2.285	0.797	2070
MAP	15693	C	T	0.018	-2.511	4.568	0.583	2070
MAP	15904	T	C	0.055	-0.431	1.07	0.687	4250
SBP	15904	T	C	0.055	-1.177	2.395	0.623	4250
DBP	15904	T	C	0.055	-0.267	0.988	0.787	4250
MAP	15907	G	A	0.015	1.796	2.653	0.499	2066
DBP	15907	G	A	0.015	1.495	1.882	0.427	2066
SBP	15907	G	A	0.015	1.633	4.879	0.738	2066
MAP	15924	G	A	0.035	0.372	1.282	0.771	4243
DBP	15924	G	A	0.035	0.535	1.164	0.646	4243
SBP	15924	G	A	0.035	-0.213	1.789	0.905	4243
DBP	15928	A	G	0.05	0.146	1.762	0.934	4249
MAP	15928	A	G	0.05	0.226	1.367	0.869	4249
SBP	15928	A	G	0.05	1.846	1.844	0.317	4249
DBP	16069	T	C	0.067	-0.752	1.069	0.482	2064
SBP	16069	T	C	0.067	0.179	1.531	0.907	2064
MAP	16069	T	C	0.067	-0.465	1.15	0.686	2064
MAP	16145	A	G	0.015	2.477	2.126	0.244	2064
DBP	16145	A	G	0.015	1.749	1.943	0.368	2064
SBP	16145	A	G	0.015	2.611	2.945	0.375	2064
SBP	16163	G	A	0.035	0.793	2.997	0.791	2056
MAP	16163	G	A	0.035	0.511	1.515	0.736	2056
DBP	16163	G	A	0.035	0.717	2.123	0.735	2056
SBP	16270	T	C	0.187	0.871	1.961	0.657	2058
DBP	16270	T	C	0.187	-0.227	1.295	0.861	2058
MAP	16270	T	C	0.187	-0.081	1.417	0.955	2058
DBP	16356	C	T	0.034	-1.242	1.227	0.311	2031
SBP	16356	C	T	0.034	-2.879	3.004	0.338	2031
MAP	16356	C	T	0.034	-1.312	1.328	0.323	2031
DBP	16399	G	A	0.036	-0.608	1.243	0.625	2056
SBP	16399	G	A	0.036	-0.385	1.849	0.835	2056
MAP	16399	G	A	0.036	-0.627	1.354	0.643	2056

Abbreviations and definitions: SBP, systolic blood pressure; DBP, diastolic blood pressure; MAP, mean arterial pressure; VAF, variant allele frequency; Beta, beta coefficient; SE, corresponding standard error to Beta, P, p-value; N, sample size

Table S2. Meta-analysis results of sex-differentiated analysis

Trait	mtDNA position	Variant allele	Reference allele	Male VAF	Male beta	Male SE	Male P	Male N	Female VAF	Female beta	Female SE	Female P	Female N	Sex-differentiated P	Heterogeneity P
SBP	93	G	A	0.023	8.284	3.203	9.72E-03	1018	0.022	-2.809	3.384	4.06E-01	1023	2.50E-02	9.13E-03
DBP	93	G	A	0.023	1.749	2.236	4.34E-01	1018	0.022	-3.841	2.174	7.73E-02	1023	1.55E-01	6.63E-02
MAP	93	G	A	0.023	3.947	2.373	9.63E-02	1018	0.022	-3.467	2.396	1.48E-01	1023	8.80E-02	2.76E-02
DBP	199	C	T	0.029	2.642	2.143	2.18E-01	1003	0.038	-0.080	2.862	9.78E-01	1020	4.68E-01	4.60E-01
SBP	199	C	T	0.029	5.824	3.096	6.00E-02	1003	0.038	2.676	3.383	4.29E-01	1020	1.25E-01	4.92E-01
MAP	199	C	T	0.029	3.900	2.280	8.72E-02	1003	0.038	1.618	2.370	4.95E-01	1020	1.83E-01	4.88E-01
DBP	217	C	T	0.054	-2.580	1.623	1.12E-01	1001	0.067	1.189	1.627	4.65E-01	1015	2.17E-01	8.73E-02
SBP	217	C	T	0.054	-2.637	2.279	2.47E-01	1001	0.067	0.886	2.177	6.84E-01	1015	4.71E-01	2.64E-01
MAP	217	C	T	0.054	-2.864	1.710	9.40E-02	1001	0.067	1.166	1.891	5.38E-01	1015	2.04E-01	8.78E-02
SBP	228	A	G	0.035	-5.625	3.144	7.36E-02	999	0.041	-0.791	3.497	8.21E-01	1017	1.97E-01	3.04E-01
DBP	228	A	G	0.035	-1.572	2.130	4.61E-01	999	0.041	-2.118	2.182	3.32E-01	1017	4.75E-01	8.58E-01
MAP	228	A	G	0.035	-2.985	2.287	1.92E-01	999	0.041	-1.736	2.431	4.75E-01	1017	3.31E-01	7.08E-01
MAP	295	T	C	0.075	-1.777	2.058	3.88E-01	989	0.064	0.115	3.722	9.75E-01	998	6.88E-01	6.38E-01
DBP	295	T	C	0.075	-1.791	1.923	3.52E-01	989	0.064	0.520	4.045	8.98E-01	998	6.43E-01	5.70E-01
SBP	295	T	C	0.075	-2.015	2.802	4.72E-01	989	0.064	-0.237	4.484	9.58E-01	998	7.71E-01	7.37E-01
DBP	456	T	C	0.026	-2.435	2.130	2.53E-01	1002	0.025	1.405	1.987	4.79E-01	1006	4.05E-01	1.87E-01
MAP	456	T	C	0.026	-1.052	2.302	6.48E-01	1002	0.025	0.887	2.192	6.86E-01	1006	8.30E-01	5.42E-01
SBP	456	T	C	0.026	1.021	3.194	7.49E-01	1002	0.025	0.386	3.106	9.01E-01	1006	9.43E-01	8.87E-01
MAP	477	C	T	0.018	1.061	4.573	8.17E-01	1003	0.018	-0.203	2.577	9.37E-01	1011	9.70E-01	8.13E-01
DBP	477	C	T	0.018	1.314	4.630	7.77E-01	1003	0.018	-1.670	2.300	4.68E-01	1011	7.38E-01	4.68E-01
SBP	477	C	T	0.018	-0.151	4.040	9.70E-01	1003	0.018	2.709	3.721	4.67E-01	1011	7.67E-01	5.35E-01
DBP	499	A	G	0.022	-2.357	2.306	3.07E-01	993	0.017	0.398	3.647	9.13E-01	1012	5.89E-01	3.37E-01
MAP	499	A	G	0.022	-3.185	2.401	1.85E-01	993	0.017	-2.086	7.077	7.68E-01	1012	3.97E-01	2.72E-01
SBP	499	A	G	0.022	-5.024	4.307	2.43E-01	993	0.017	-6.343	13.663	6.42E-01	1012	4.55E-01	4.51E-01
MAP	709	A	G	0.097	2.471	1.619	1.27E-01	1013	0.118	-2.480	1.590	1.19E-01	1026	9.24E-02	2.91E-02
SBP	709	A	G	0.097	0.147	2.410	9.51E-01	1013	0.118	-4.631	2.322	4.61E-02	1026	1.37E-01	1.10E-01
DBP	709	A	G	0.097	3.613	1.496	1.58E-02	1013	0.118	-1.434	1.416	3.11E-01	1026	3.24E-02	1.11E-02
MAP	951	A	G	0.031	1.015	2.103	6.30E-01	1010	0.032	-1.842	1.907	3.34E-01	1022	5.58E-01	3.14E-01
SBP	951	A	G	0.031	1.956	3.382	5.63E-01	1010	0.032	-0.499	2.704	8.53E-01	1022	8.32E-01	5.83E-01
DBP	951	A	G	0.031	0.120	1.987	9.52E-01	1010	0.032	-1.853	2.139	3.86E-01	1022	6.86E-01	8.32E-01
SBP	1243	C	T	0.023	-2.133	2.947	4.69E-01	2363	0.025	-6.213	4.373	1.55E-01	1856	2.80E-01	4.39E-01
MAP	1243	C	T	0.023	0.477	2.654	8.57E-01	2363	0.025	-2.702	2.918	3.55E-01	1856	6.41E-01	3.88E-01
DBP	1243	C	T	0.023	2.060	2.878	4.74E-01	2363	0.025	-1.152	2.559	6.53E-01	1856	6.99E-01	4.85E-01
SBP	1719	A	G	0.046	0.802	2.329	7.31E-01	2365	0.051	1.890	2.709	4.85E-01	1846	7.39E-01	5.96E-01
DBP	1719	A	G	0.046	-0.422	1.487	7.76E-01	2365	0.051	-0.896	1.689	5.96E-01	1846	8.35E-01	5.49E-01

MAP	1719	A	G	0.046	0.160	1.729	9.26E-01	2365	0.051	-0.118	1.884	9.50E-01	1846	9.94E-01	8.69E-01
DBP	1811	G	A	0.086	-0.442	1.106	6.89E-01	2326	0.081	2.024	1.381	1.43E-01	1846	3.15E-01	1.63E-01
SBP	1811	G	A	0.086	-1.090	1.758	5.35E-01	2326	0.081	1.714	2.485	4.90E-01	1846	6.50E-01	3.57E-01
MAP	1811	G	A	0.086	-0.749	1.219	5.39E-01	2326	0.081	1.864	1.605	2.46E-01	1846	4.22E-01	1.95E-01
MAP	1888	A	G	0.049	1.823	2.355	4.39E-01	2359	0.054	-0.612	5.632	9.13E-01	1843	7.37E-01	6.12E-01
DBP	1888	A	G	0.049	1.960	2.166	3.66E-01	2359	0.054	-0.668	5.835	9.09E-01	1843	6.60E-01	6.68E-01
SBP	1888	A	G	0.049	1.788	3.321	5.90E-01	2359	0.054	0.652	5.808	9.11E-01	1843	8.60E-01	8.48E-01
MAP	2706	G	A	0.562	1.501	1.240	2.26E-01	2369	0.554	-0.348	2.144	8.71E-01	1854	4.74E-01	4.55E-01
SBP	2706	G	A	0.562	1.044	1.781	5.58E-01	2369	0.554	-0.403	3.237	9.01E-01	1854	8.36E-01	6.95E-01
DBP	2706	G	A	0.562	1.773	1.127	1.16E-01	2369	0.554	0.082	1.874	9.65E-01	1854	2.90E-01	4.39E-01
MAP	3010	A	G	0.272	0.113	1.002	9.10E-01	1015	0.278	-0.576	1.153	6.17E-01	1029	8.77E-01	6.52E-01
DBP	3010	A	G	0.272	0.188	0.932	8.40E-01	1015	0.278	-0.575	1.029	5.76E-01	1029	8.38E-01	5.83E-01
SBP	3010	A	G	0.272	0.086	1.382	9.50E-01	1015	0.278	-0.526	1.675	7.54E-01	1029	9.50E-01	7.78E-01
DBP	3197	C	T	0.194	0.258	2.063	9.00E-01	1013	0.209	-0.992	3.624	7.84E-01	1030	9.56E-01	7.72E-01
MAP	3197	C	T	0.194	-0.507	2.234	8.20E-01	1013	0.209	0.115	2.674	9.66E-01	1030	9.74E-01	8.58E-01
SBP	3197	C	T	0.194	-2.605	3.116	4.03E-01	1013	0.209	1.174	3.875	7.62E-01	1030	6.74E-01	4.47E-01
MAP	3480	G	A	0.051	2.243	3.004	4.55E-01	1834	0.044	-3.888	3.660	2.88E-01	1474	4.30E-01	1.95E-01
DBP	3480	G	A	0.051	0.967	2.833	7.33E-01	1834	0.044	-3.100	3.324	3.51E-01	1474	6.11E-01	3.52E-01
SBP	3480	G	A	0.051	4.060	4.034	3.14E-01	1834	0.044	-6.391	5.147	2.14E-01	1474	2.79E-01	1.10E-01
MAP	4024	G	A	0.014	5.406	3.034	7.48E-02	1016	0.017	4.480	5.228	3.91E-01	1024	1.42E-01	6.97E-01
SBP	4024	G	A	0.014	3.560	4.098	3.85E-01	1016	0.017	8.221	11.836	4.87E-01	1024	5.39E-01	8.55E-01
DBP	4024	G	A	0.014	6.794	2.857	1.74E-02	1016	0.017	2.138	2.367	3.66E-01	1024	3.93E-02	2.09E-01
MAP	4216	C	T	0.113	-2.441	3.562	4.93E-01	1004	0.117	3.994	4.645	3.90E-01	1028	5.46E-01	2.72E-01
SBP	4216	C	T	0.113	-1.268	4.897	7.96E-01	1004	0.117	3.097	6.643	6.41E-01	1028	8.67E-01	5.97E-01
DBP	4216	C	T	0.113	-3.012	3.321	3.64E-01	1004	0.117	4.312	4.185	3.03E-01	1028	3.90E-01	1.70E-01
MAP	4769	G	A	0.958	-1.288	1.821	4.79E-01	1021	0.958	-0.414	1.681	8.06E-01	1037	7.55E-01	7.24E-01
DBP	4769	G	A	0.958	-0.246	1.715	8.86E-01	1021	0.958	0.016	1.521	9.92E-01	1037	9.90E-01	9.09E-01
SBP	4769	G	A	0.958	-3.153	3.862	4.14E-01	1021	0.958	-2.136	2.393	3.72E-01	1037	4.81E-01	5.96E-01
MAP	4917	G	A	0.043	1.828	2.590	4.80E-01	1010	0.054	2.681	3.370	4.26E-01	1022	5.68E-01	8.41E-01
DBP	4917	G	A	0.043	1.383	2.395	5.64E-01	1010	0.054	1.954	2.925	5.04E-01	1022	6.77E-01	8.80E-01
SBP	4917	G	A	0.043	3.237	3.613	3.70E-01	1010	0.054	4.321	5.163	4.03E-01	1022	4.72E-01	8.63E-01
DBP	5004	C	T	0.014	6.797	2.860	1.75E-02	1014	0.017	2.092	2.369	3.77E-01	1021	4.02E-02	2.05E-01
MAP	5004	C	T	0.014	5.434	3.039	7.37E-02	1014	0.017	4.437	5.295	4.02E-01	1021	1.42E-01	8.36E-01
SBP	5004	C	T	0.014	3.638	4.097	3.75E-01	1014	0.017	8.171	11.914	4.93E-01	1021	5.33E-01	7.99E-01
DBP	5263	T	C	0.020	0.082	2.404	9.73E-01	1013	0.021	4.270	2.337	6.77E-02	1021	1.88E-01	2.12E-01
MAP	5263	T	C	0.020	-1.490	2.567	5.62E-01	1013	0.021	3.850	2.588	1.37E-01	1021	2.79E-01	1.43E-01
SBP	5263	T	C	0.020	-5.069	3.496	1.47E-01	1013	0.021	2.403	3.684	5.14E-01	1021	2.83E-01	1.41E-01

MAP	5390	G	A	0.017	-1.599	2.743	5.60E-01	1017	0.018	1.718	3.152	5.86E-01	1024	7.27E-01	4.26E-01
SBP	5390	G	A	0.017	-2.399	3.626	5.08E-01	1017	0.018	1.824	3.975	6.46E-01	1024	7.23E-01	4.33E-01
DBP	5390	G	A	0.017	-1.370	2.619	6.01E-01	1017	0.018	1.711	2.381	4.72E-01	1024	6.74E-01	3.84E-01
MAP	5460	A	G	0.041	3.550	2.529	1.60E-01	1012	0.036	-0.611	2.766	8.25E-01	1021	3.64E-01	2.67E-01
DBP	5460	A	G	0.041	4.866	2.293	3.39E-02	1012	0.036	1.233	2.438	6.13E-01	1021	9.27E-02	2.78E-01
SBP	5460	A	G	0.041	1.258	3.651	7.30E-01	1012	0.036	-5.204	4.107	2.05E-01	1021	4.22E-01	2.40E-01
SBP	7768	G	A	0.114	2.214	2.539	3.83E-01	1009	0.133	-1.195	3.215	7.10E-01	1025	6.38E-01	4.05E-01
DBP	7768	G	A	0.114	-0.288	1.703	8.66E-01	1009	0.133	0.571	3.034	8.51E-01	1025	9.68E-01	8.01E-01
MAP	7768	G	A	0.114	0.411	1.830	8.22E-01	1009	0.133	0.119	2.664	9.64E-01	1025	9.74E-01	8.78E-01
SBP	8869	G	A	0.022	-4.572	4.178	2.74E-01	1027	0.024	3.190	3.482	3.60E-01	1042	3.61E-01	1.60E-01
MAP	8869	G	A	0.022	-1.115	2.405	6.43E-01	1027	0.024	3.607	2.434	1.38E-01	1042	2.99E-01	1.68E-01
DBP	8869	G	A	0.022	0.338	2.248	8.80E-01	1027	0.024	3.609	2.196	1.00E-01	1042	2.56E-01	2.98E-01
MAP	9055	A	G	0.055	1.532	2.409	5.25E-01	1845	0.052	1.304	2.517	6.04E-01	1489	7.14E-01	9.48E-01
SBP	9055	A	G	0.055	2.184	3.300	5.08E-01	1845	0.052	-0.501	3.606	8.89E-01	1489	7.96E-01	5.83E-01
DBP	9055	A	G	0.055	1.067	2.247	6.35E-01	1845	0.052	1.745	2.258	4.40E-01	1489	6.63E-01	8.31E-01
SBP	9477	A	G	0.185	-1.981	3.331	5.52E-01	1023	0.193	1.036	4.190	8.05E-01	1039	8.13E-01	5.73E-01
MAP	9477	A	G	0.185	0.536	2.349	8.20E-01	1023	0.193	-0.162	2.853	9.55E-01	1039	9.73E-01	8.50E-01
DBP	9477	A	G	0.185	1.512	2.152	4.82E-01	1023	0.193	-1.043	3.305	7.52E-01	1039	7.43E-01	4.72E-01
MAP	9667	G	A	0.021	0.925	2.635	7.26E-01	1024	0.021	-2.975	2.898	3.05E-01	1045	5.55E-01	3.65E-01
DBP	9667	G	A	0.021	1.166	2.424	6.30E-01	1024	0.021	-4.787	3.981	2.29E-01	1045	4.32E-01	2.87E-01
SBP	9667	G	A	0.021	-0.050	3.719	9.89E-01	1024	0.021	-0.363	3.350	9.14E-01	1045	9.94E-01	9.50E-01
DBP	9698	C	T	0.064	-0.452	1.387	7.45E-01	2384	0.052	-1.231	1.913	5.20E-01	1875	7.71E-01	7.56E-01
SBP	9698	C	T	0.064	0.882	2.198	6.88E-01	2384	0.052	-2.866	4.270	5.02E-01	1875	7.37E-01	4.68E-01
MAP	9698	C	T	0.064	0.005	1.526	9.97E-01	2384	0.052	-1.816	2.493	4.66E-01	1875	7.67E-01	5.96E-01
DBP	9899	C	T	0.010	2.813	2.676	2.93E-01	1898	0.013	1.414	2.801	6.14E-01	1489	5.07E-01	7.49E-01
SBP	9899	C	T	0.010	1.043	4.706	8.25E-01	1898	0.013	0.970	3.961	8.06E-01	1489	9.47E-01	9.90E-01
MAP	9899	C	T	0.010	2.297	3.045	4.51E-01	1898	0.013	1.135	2.728	6.77E-01	1489	6.90E-01	7.76E-01
MAP	9903	C	T	0.023	-2.621	2.564	3.07E-01	1028	0.021	-3.509	2.934	2.32E-01	1043	2.90E-01	8.20E-01
DBP	9903	C	T	0.023	-1.489	2.361	5.28E-01	1028	0.021	-1.844	2.635	4.84E-01	1043	6.42E-01	9.20E-01
SBP	9903	C	T	0.023	-5.487	3.609	1.28E-01	1028	0.021	-7.217	4.228	8.78E-02	1043	7.33E-02	7.56E-01
SBP	10034	C	T	0.026	5.906	4.903	2.28E-01	2383	0.024	-0.358	4.319	9.34E-01	1869	4.82E-01	7.07E-01
MAP	10034	C	T	0.026	3.840	3.707	3.00E-01	2383	0.024	-1.540	2.995	6.07E-01	1869	5.12E-01	3.81E-01
DBP	10034	C	T	0.026	2.402	3.012	4.25E-01	2383	0.024	-2.312	2.686	3.89E-01	1869	5.02E-01	2.51E-01
MAP	10238	C	T	0.028	3.716	3.255	2.54E-01	2385	0.028	-3.368	3.931	3.92E-01	1873	3.61E-01	1.75E-01
SBP	10238	C	T	0.028	5.263	4.036	1.92E-01	2385	0.028	-1.438	4.249	7.35E-01	1873	4.04E-01	3.10E-01
DBP	10238	C	T	0.028	2.546	2.778	3.59E-01	2385	0.028	-3.492	3.340	2.96E-01	1873	3.80E-01	1.66E-01
DBP	10463	C	T	0.049	1.438	2.059	4.85E-01	2387	0.057	4.054	2.414	9.31E-02	1875	1.91E-01	4.10E-01

MAP	10463	C	T	0.049	0.713	2.243	7.50E-01	2387	0.057	4.118	2.752	1.35E-01	1875	3.10E-01	3.38E-01
SBP	10463	C	T	0.049	-0.154	3.170	9.61E-01	2387	0.057	3.354	4.129	4.17E-01	1875	7.18E-01	5.00E-01
DBP	10550	G	A	0.053	-0.157	1.633	9.24E-01	2387	0.043	-0.715	2.104	7.34E-01	1875	9.40E-01	8.34E-01
SBP	10550	G	A	0.053	2.487	2.631	3.44E-01	2387	0.043	-4.528	3.636	2.13E-01	1875	2.95E-01	1.18E-01
MAP	10550	G	A	0.053	0.727	1.806	6.87E-01	2387	0.043	-1.842	2.408	4.44E-01	1875	6.88E-01	3.93E-01
SBP	11299	C	T	0.051	5.494	4.570	2.29E-01	1848	0.044	-3.645	4.857	4.53E-01	1491	3.66E-01	1.88E-01
MAP	11299	C	T	0.051	3.107	2.892	2.83E-01	1848	0.044	-2.274	3.459	5.11E-01	1491	4.52E-01	2.33E-01
DBP	11299	C	T	0.051	1.676	2.724	5.38E-01	1848	0.044	-1.858	3.155	5.56E-01	1491	6.96E-01	3.97E-01
MAP	11467	G	A	0.293	-2.341	2.418	3.33E-01	1025	0.273	4.486	4.515	3.20E-01	1041	3.82E-01	1.70E-01
DBP	11467	G	A	0.293	-1.878	2.252	4.04E-01	1025	0.273	5.008	4.671	2.84E-01	1041	3.98E-01	2.04E-01
SBP	11467	G	A	0.293	-3.558	3.329	2.85E-01	1025	0.273	4.105	4.949	4.07E-01	1041	4.01E-01	1.88E-01
SBP	11674	T	C	0.029	-3.449	2.976	2.47E-01	1847	0.029	10.518	16.974	5.35E-01	1495	4.22E-01	1.99E-01
MAP	11674	T	C	0.029	-3.643	2.007	6.95E-02	1847	0.029	3.125	4.179	4.55E-01	1495	1.46E-01	6.64E-02
DBP	11674	T	C	0.029	-3.456	1.793	5.40E-02	1847	0.029	2.624	3.415	4.42E-01	1495	1.16E-01	8.87E-02
MAP	11812	G	A	0.041	-0.328	2.496	8.96E-01	1848	0.039	-1.543	2.798	5.81E-01	1493	8.52E-01	7.46E-01
DBP	11812	G	A	0.041	0.243	2.292	9.15E-01	1848	0.039	-1.577	2.489	5.26E-01	1493	8.13E-01	5.91E-01
SBP	11812	G	A	0.041	-2.173	3.509	5.36E-01	1848	0.039	-1.796	4.058	6.58E-01	1493	7.49E-01	9.44E-01
DBP	11899	C	T	0.012	5.554	1.998	5.44E-03	2379	0.013	0.994	5.303	8.51E-01	1487	2.06E-02	1.86E-02
MAP	11899	C	T	0.012	6.916	2.183	1.54E-03	2379	0.013	2.305	6.715	7.31E-01	1487	6.23E-03	8.33E-03
SBP	11899	C	T	0.012	9.777	3.096	1.60E-03	2379	0.013	4.116	8.908	6.44E-01	1487	6.15E-03	1.42E-02
SBP	11914	A	G	0.040	0.275	1.784	8.78E-01	2373	0.047	0.763	2.158	7.24E-01	1875	9.28E-01	8.62E-01
DBP	11914	A	G	0.040	0.299	1.127	7.91E-01	2373	0.047	1.812	1.455	2.13E-01	1875	4.45E-01	4.66E-01
MAP	11914	A	G	0.040	0.395	1.239	7.50E-01	2373	0.047	1.661	1.744	3.41E-01	1875	6.04E-01	7.25E-01
MAP	12308	G	A	0.304	-0.272	1.981	8.91E-01	2377	0.293	1.576	5.115	7.58E-01	1869	9.45E-01	8.39E-01
SBP	12308	G	A	0.304	-2.476	2.799	3.76E-01	2377	0.293	0.981	7.097	8.90E-01	1869	6.70E-01	4.51E-01
DBP	12308	G	A	0.304	0.672	2.179	7.58E-01	2377	0.293	2.179	4.430	6.23E-01	1869	8.45E-01	7.03E-01
MAP	12372	A	G	0.307	0.968	2.822	7.32E-01	1842	0.300	-5.064	15.601	7.46E-01	1486	8.95E-01	6.73E-01
DBP	12372	A	G	0.307	2.471	2.441	3.12E-01	1842	0.300	-4.010	14.244	7.78E-01	1486	5.76E-01	3.41E-01
SBP	12372	A	G	0.307	-2.270	3.298	4.91E-01	1842	0.300	-5.504	18.793	7.70E-01	1486	7.56E-01	4.60E-01
DBP	12612	G	A	0.063	0.413	1.537	7.88E-01	1847	0.068	7.532	3.840	4.98E-02	1495	1.41E-01	8.07E-02
SBP	12612	G	A	0.063	1.058	2.390	6.58E-01	1847	0.068	7.614	5.835	1.92E-01	1495	3.87E-01	2.98E-01
MAP	12612	G	A	0.063	0.670	1.682	6.91E-01	1847	0.068	7.583	4.188	7.02E-02	1495	1.79E-01	1.20E-01
DBP	13708	A	G	0.072	-0.898	1.526	5.56E-01	2336	0.069	1.288	4.126	7.55E-01	1845	8.01E-01	5.06E-01
MAP	13708	A	G	0.072	-1.094	1.638	5.04E-01	2336	0.069	1.632	4.414	7.12E-01	1845	7.47E-01	4.45E-01
SBP	13708	A	G	0.072	-1.678	2.134	4.32E-01	2336	0.069	3.064	5.100	5.48E-01	1845	6.13E-01	3.25E-01
DBP	13759	A	G	0.024	-1.443	3.642	6.92E-01	1021	0.028	1.135	3.068	7.11E-01	1036	8.63E-01	5.88E-01
MAP	13759	A	G	0.024	-0.627	4.173	8.81E-01	1021	0.028	1.133	2.560	6.58E-01	1036	8.96E-01	6.69E-01

SBP	13759	A	G	0.024	1.572	6.031	7.94E-01	1021	0.028	1.516	2.906	6.02E-01	1036	8.44E-01	9.01E-01
DBP	13928	C	G	0.024	-1.087	1.641	5.08E-01	1838	0.028	3.437	1.685	4.13E-02	1481	1.00E-01	4.99E-02
SBP	13928	C	G	0.024	-5.731	2.506	2.22E-02	1838	0.028	5.978	4.093	1.44E-01	1481	2.52E-02	6.67E-03
MAP	13928	C	G	0.024	-2.593	1.785	1.46E-01	1838	0.028	4.164	1.897	2.82E-02	1481	3.13E-02	9.19E-03
SBP	13966	G	A	0.015	-2.367	4.941	6.32E-01	2382	0.020	2.929	3.582	4.14E-01	1871	6.38E-01	3.45E-01
MAP	13966	G	A	0.015	-2.684	2.507	2.84E-01	2382	0.020	0.691	2.666	7.96E-01	1871	5.45E-01	3.89E-01
DBP	13966	G	A	0.015	-2.887	1.797	1.08E-01	2382	0.020	0.255	2.702	9.25E-01	1871	2.74E-01	2.87E-01
MAP	14182	C	T	0.126	2.085	1.770	2.39E-01	1014	0.141	0.413	2.869	8.86E-01	1038	4.94E-01	4.90E-01
DBP	14182	C	T	0.126	1.221	1.636	4.55E-01	1014	0.141	0.675	2.887	8.15E-01	1038	7.36E-01	8.56E-01
SBP	14182	C	T	0.126	3.727	2.470	1.31E-01	1014	0.141	-0.780	3.340	8.15E-01	1038	3.12E-01	2.44E-01
MAP	14233	G	A	0.035	0.938	2.630	7.22E-01	1026	0.049	1.459	3.434	6.71E-01	1045	8.57E-01	7.78E-01
SBP	14233	G	A	0.035	0.174	4.955	9.72E-01	1026	0.049	1.920	3.481	5.81E-01	1045	8.58E-01	6.97E-01
DBP	14233	G	A	0.035	1.370	2.438	5.74E-01	1026	0.049	0.945	3.262	7.72E-01	1045	8.19E-01	8.13E-01
SBP	14766	T	C	0.509	-5.059	3.831	1.87E-01	1841	0.504	11.745	5.530	3.37E-02	1487	4.38E-02	1.27E-02
MAP	14766	T	C	0.509	-4.454	2.653	9.32E-02	1841	0.504	5.642	3.969	1.55E-01	1487	8.89E-02	2.83E-02
DBP	14766	T	C	0.509	-4.483	2.401	6.19E-02	1841	0.504	3.190	3.634	3.80E-01	1487	1.19E-01	4.61E-02
DBP	14905	A	G	0.052	4.010	4.741	3.98E-01	1802	0.056	3.077	14.783	8.35E-01	1470	6.84E-01	5.33E-01
MAP	14905	A	G	0.052	2.038	4.165	6.25E-01	1802	0.056	4.425	14.458	7.60E-01	1470	8.47E-01	7.76E-01
SBP	14905	A	G	0.052	-0.973	4.748	8.38E-01	1802	0.056	4.952	14.002	7.24E-01	1470	9.20E-01	6.95E-01
SBP	15043	A	G	0.038	2.786	3.731	4.55E-01	2372	0.040	0.461	2.742	8.66E-01	1868	7.46E-01	7.97E-01
DBP	15043	A	G	0.038	1.697	2.589	5.12E-01	2372	0.040	-1.164	1.718	4.98E-01	1868	6.41E-01	3.78E-01
MAP	15043	A	G	0.038	2.257	2.992	4.51E-01	2372	0.040	-0.556	1.912	7.71E-01	1868	7.21E-01	5.91E-01
DBP	15218	G	A	0.024	-0.406	2.259	8.57E-01	1022	0.027	-0.871	1.911	6.48E-01	1040	8.87E-01	8.75E-01
MAP	15218	G	A	0.024	-1.440	2.429	5.53E-01	1022	0.027	-0.841	2.117	6.91E-01	1040	7.75E-01	8.52E-01
SBP	15218	G	A	0.024	-3.596	3.356	2.84E-01	1022	0.027	-0.668	3.024	8.25E-01	1040	5.50E-01	5.17E-01
MAP	15452	A	C	0.115	-1.263	3.316	7.03E-01	2382	0.118	14.909	5.336	5.22E-03	1872	1.88E-02	7.62E-03
DBP	15452	A	C	0.115	-0.729	2.795	7.94E-01	2382	0.118	12.181	4.737	1.01E-02	1872	3.54E-02	1.46E-02
SBP	15452	A	C	0.115	-1.685	4.440	7.04E-01	2382	0.118	19.008	7.831	1.52E-02	1872	4.89E-02	1.92E-02
DBP	15607	G	A	0.051	2.886	4.611	5.31E-01	1847	0.054	-9.143	6.291	1.46E-01	1491	2.86E-01	1.14E-01
MAP	15607	G	A	0.051	1.215	3.889	7.55E-01	1847	0.054	-7.774	6.883	2.59E-01	1491	5.03E-01	2.52E-01
SBP	15607	G	A	0.051	-1.255	4.381	7.75E-01	1847	0.054	-6.011	9.627	5.32E-01	1491	7.90E-01	6.53E-01
MAP	15904	T	C	0.055	1.371	1.063	1.97E-01	2382	0.055	-0.349	2.182	8.73E-01	1868	4.30E-01	4.79E-01
DBP	15904	T	C	0.055	1.396	0.962	1.47E-01	2382	0.055	0.261	1.945	8.93E-01	1868	3.46E-01	6.01E-01
SBP	15904	T	C	0.055	0.900	1.971	6.48E-01	2382	0.055	-2.165	3.182	4.96E-01	1868	7.15E-01	4.33E-01
SBP	15924	G	A	0.035	0.461	2.051	8.22E-01	2375	0.035	-3.273	3.172	3.02E-01	1868	5.73E-01	3.23E-01
MAP	15924	G	A	0.035	-0.091	1.410	9.49E-01	2375	0.035	-2.155	2.168	3.20E-01	1868	6.09E-01	4.25E-01
DBP	15924	G	A	0.035	-0.411	1.273	7.47E-01	2375	0.035	-1.594	1.925	4.08E-01	1868	6.74E-01	6.08E-01

SBP	15928	A	G	0.048	0.262	3.429	9.39E-01	2379	0.053	1.734	5.407	7.48E-01	1870	9.47E-01	8.18E-01
DBP	15928	A	G	0.048	1.584	2.483	5.24E-01	2379	0.053	-2.157	6.394	7.36E-01	1870	7.71E-01	6.12E-01
MAP	15928	A	G	0.048	0.819	2.400	7.33E-01	2379	0.053	-0.734	5.812	9.00E-01	1870	9.36E-01	7.41E-01
MAP	16069	T	C	0.073	-1.568	2.027	4.39E-01	1025	0.062	2.764	3.243	3.94E-01	1039	5.16E-01	2.53E-01
DBP	16069	T	C	0.073	-1.456	1.893	4.42E-01	1025	0.062	2.741	3.652	4.53E-01	1039	5.61E-01	2.83E-01
SBP	16069	T	C	0.073	-2.182	2.778	4.32E-01	1025	0.062	3.181	4.433	4.73E-01	1039	5.68E-01	3.05E-01
SBP	16270	T	C	0.181	-0.534	3.613	8.82E-01	1023	0.192	2.609	3.493	4.55E-01	1035	7.48E-01	4.91E-01
DBP	16270	T	C	0.181	0.319	1.904	8.67E-01	1023	0.192	0.886	2.542	7.27E-01	1035	9.28E-01	8.54E-01
MAP	16270	T	C	0.181	0.012	2.348	9.96E-01	1023	0.192	1.723	2.403	4.73E-01	1035	7.73E-01	6.11E-01
SBP	16356	C	T	0.041	-2.194	2.405	3.62E-01	1008	0.028	-3.323	5.135	5.18E-01	1023	5.35E-01	6.98E-01
MAP	16356	C	T	0.041	-2.216	1.789	2.15E-01	1008	0.028	-1.581	2.562	5.37E-01	1023	3.84E-01	6.76E-01
DBP	16356	C	T	0.041	-2.186	1.691	1.96E-01	1008	0.028	-0.932	1.808	6.06E-01	1023	3.80E-01	6.12E-01
SBP	16399	G	A	0.035	-0.615	2.744	8.23E-01	1021	0.036	-0.454	2.662	8.65E-01	1035	9.61E-01	9.66E-01
DBP	16399	G	A	0.035	-0.655	1.874	7.27E-01	1021	0.036	-0.926	1.669	5.79E-01	1035	8.06E-01	9.14E-01
MAP	16399	G	A	0.035	-0.889	2.005	6.57E-01	1021	0.036	-0.632	1.854	7.33E-01	1035	8.55E-01	9.25E-01

Abbreviations and definitions: SBP, systolic blood pressure; DBP, diastolic blood pressure; MAP, mean arterial pressure; VAF, variant allele frequency; Beta, beta coefficient; SE, corresponding standard error to Beta, P, p-value; N, sample size

Table S3. Results from SKAT meta-analysis across seven mtDNA regions

mtDNA region	Trait	Qmeta	cMAF	mtSNPs	P	T
Complex I	SBP	14133	0.810	332	0.105	T1
Complex III	SBP	3015	0.159	70	0.314	T1
Complex IV	SBP	5788	0.339	148	0.193	T1
Complex V	SBP	3555	0.150	69	0.025	T1
Control region	SBP	6261	0.476	169	0.456	T1
rRNA	SBP	1966	0.126	60	0.370	T1
tRNA	SBP	2107	0.111	67	0.124	T1
Complex I	MAP	25819	0.810	332	0.082	T1
Complex III	MAP	6732	0.159	70	0.156	T1
Complex IV	MAP	10515	0.339	148	0.166	T1
Complex V	MAP	5786	0.150	69	0.041	T1
Control region	MAP	12223	0.476	169	0.214	T1
rRNA	MAP	4118	0.126	60	0.153	T1
tRNA	MAP	3274	0.111	67	0.250	T1
Complex I	DBP	28458	0.810	332	0.091	T1
Complex III	DBP	7447	0.159	70	0.179	T1
Complex IV	DBP	11728	0.339	148	0.157	T1
Complex V	DBP	5370	0.150	69	0.118	T1
Control region	DBP	13908	0.476	169	0.148	T1
rRNA	DBP	4701	0.126	60	0.123	T1
tRNA	DBP	3268	0.111	67	0.382	T1
Complex I	SBP	23587	2.371	402	0.175	T5
Complex III	SBP	3895	0.331	77	0.366	T5
Complex IV	SBP	8715	0.718	169	0.223	T5
Complex V	SBP	4921	0.434	85	0.200	T5
Control region	SBP	9074	1.622	214	0.891	T5
rRNA	SBP	5205	0.468	73	0.053	T5
tRNA	SBP	3020	0.273	75	0.302	T5
Complex I	MAP	41026	2.371	402	0.243	T5
Complex III	MAP	7863	0.331	77	0.267	T5
Complex IV	MAP	14509	0.718	169	0.342	T5
Complex V	MAP	7832	0.434	85	0.326	T5
Control region	MAP	20319	1.622	214	0.434	T5
rRNA	MAP	8919	0.468	73	0.089	T5
tRNA	MAP	5579	0.273	75	0.295	T5
Complex I	DBP	45922	2.371	402	0.244	T5
Complex III	DBP	8340	0.331	77	0.354	T5
Complex IV	DBP	15944	0.718	169	0.359	T5
Complex V	DBP	7994	0.434	85	0.434	T5
Control region	DBP	24921	1.622	214	0.212	T5
rRNA	DBP	8547	0.468	73	0.241	T5
tRNA	DBP	5941	0.273	75	0.372	T5

Abbreviations and definitions: mtDNA region, one of the tested mtDNA regions; Trait, the trait used for a specific test; cMAF, cumulative minor allele frequency; mtSNPs, the number of mtSNPs used in the analysis; Qmeta, SKAT Q statistics: Qmeta=∑w^2U^2 where w is a weight for mtSNP and U is the associated score statistics; P, p-value for SKAT meta-analysis; T, MAF value threshold for selecting mtSNPs to be included in the analysis

Table S4. Results from SKAT meta-analysis across six mtDNA regions with possibly and likely pathogenic variants

mtDNA region	Trait	Qmeta	cMAF	mtSNPs	P	T
Complex I	SBP	2430	0.119	47	0.093	T1
Complex III	SBP	331	0.019	12	0.437	T1
Complex IV	SBP	874	0.045	23	0.214	T1
Complex V	SBP	1176	0.064	27	0.164	T1
Control region	SBP	1	0.002	2	0.883	T1
tRNA	SBP	38	0.004	4	0.475	T1
Complex I	MAP	5169	0.119	47	0.038	T1
Complex III	MAP	1066	0.019	12	0.088	T1
Complex IV	MAP	1191	0.045	23	0.463	T1
Complex V	MAP	1786	0.064	27	0.270	T1
Control region	MAP	5	0.002	2	0.753	T1
tRNA	MAP	51	0.004	4	0.556	T1
Complex I	DBP	5745	0.119	47	0.047	T1
Complex III	DBP	1345	0.019	12	0.056	T1
Complex IV	DBP	1112	0.045	23	0.637	T1
Complex V	DBP	1554	0.064	27	0.502	T1
Control region	DBP	15	0.002	2	0.590	T1
tRNA	DBP	47	0.004	4	0.605	T1
Complex I	SBP	4270	0.326	56	0.038	T5
Complex III	SBP	450	0.033	13	0.420	T5
Complex IV	SBP	1181	0.120	26	0.339	T5
Complex V	SBP	1240	0.110	30	0.472	T5
Control region	SBP	1	0.002	2	0.883	T5
tRNA	SBP	38	0.004	4	0.475	T5
Complex I	MAP	7244	0.326	56	0.069	T5
Complex III	MAP	1314	0.033	13	0.173	T5
Complex IV	MAP	1527	0.120	26	0.663	T5
Complex V	MAP	1936	0.110	30	0.592	T5
Control region	MAP	5	0.002	2	0.753	T5
tRNA	MAP	51	0.004	4	0.556	T5
Complex I	DBP	7651	0.326	56	0.107	T5
Complex III	DBP	1609	0.033	13	0.148	T5
Complex IV	DBP	1299	0.120	26	0.861	T5
Complex V	DBP	1844	0.110	30	0.726	T5
Control region	DBP	15	0.002	2	0.590	T5
tRNA	DBP	47	0.004	4	0.605	T5

Abbreviations and definitions: mtDNA region, one of the tested mtDNA regions; Trait, the trait used for a specific test; cMAF, cumulative minor allele frequency; mtSNPs, the number of mtSNPs used in the analysis; Qmeta, SKAT Q statistics: Qmeta=∑w^2U^2 where w is a weight for mtSNP and U is the associated score statistics; P, p-value for SKAT meta-analysis; T, MAF value threshold for selecting mtSNPs to be included in the analysis
